# *Toxoplasma gondii*-induced host cellular cell cycle dysregulation is linked to chromosome missegregation and cytokinesis failure in primary endothelial host cells

**DOI:** 10.1038/s41598-019-48961-0

**Published:** 2019-08-29

**Authors:** Zahady D. Velásquez, Iván Conejeros, Camilo Larrazabal, Katharina Kerner, Carlos Hermosilla, Anja Taubert

**Affiliations:** 1 Institute of Parasitology, Biomedical Research Center Seltersberg, Justus Liebig University Giessen, Giessen, Germany; 2 Institute for Hygiene and Infectious Diseases of Animals, Justus-Liebig-University, Giessen, Germany

**Keywords:** Chromosome segregation, Cytokinesis, Mitotic spindle

## Abstract

*Toxoplasma gondii* is a zoonotic and intracellular parasite with fast proliferating properties leading to rapid host cell lysis. *T. gondii* modulates its host cell on numerous functional levels. *T. gondii* was previously reported to influence host cellular cell cycle and to dampen host cell division. By using primary endothelial host cells, we show for the first time that *T. gondii* tachyzoite infections led to increased host cell proliferation and to an enhanced number of multi-nucleated host cells. As detected on DNA content level, parasite infections induced a G2/M cell cycle arrest without affecting expression of G2-specific cyclin B1. In line, parasite-driven impairment mainly concerned mitotic phase of host cells by propagating several functional alterations, such as chromosome segregation errors, mitotic spindle alteration and blockage of cytokinesis progression, with the latter most likely being mediated by the downregulation of the Aurora B kinase expression.

## Introduction

*Toxoplasma gondii* is a globally occurring parasite which causes severe health problems in both, humans and animals. Especially in humans and sheep, prenatal infections may lead to abortion or severely affect the progeny welfare^1,2^. In immunocompromised patients, acute *T. gondii* infections may become life threatening and recent investigations postulate a correlation between latent *T. gondii* infections and neurological/psychiatric disorders in humans^3–8^.

*T. gondii* has developed an extraordinary level of host adaptation and is capable to disrupt the host immune system and establish a life-long chronic infection^9,10^. As an obligate intracellular parasite, *T. gondii* manipulates a broad range of host cellular functions to guarantee its intracellular development and replication. Thus, it is able to reprogram the host cellular gene expression^11^ and to alter host cell division. As such, some studies report that *T. gondii* infections cause diminished host cell proliferation and host cell cycle arrest^12–14^. However, published cell cycle-related data appear inconsistent. Thus, different modes of action are recorded indicating both, an infection-driven shift from G0/G1 to S phase with accumulation of host cells in S phase^13,14^ and a host cellular arrest in G2/M phase^12^, thereby most probably reflecting cell type-specific reactions. Thus, stasis of *T. gondii*-infected human foreskin fibroblasts during S phase at G2/M border was accompanied by a delayed or missing increase of cyclins A and B in combination with an early elevation of cyclin E_1_ levels^14^. In contrast, *T. gondii*-triggered G2-arrest in human dermal fibroblasts or in a human trophoblast cell line was supported by a diminished abundance of cyclin B_1_ whilst G2/M checkpoint-related molecules were not affected^12^. In addition, Lavine and Arrizabalaga^13^ described an enhanced progression of non-infected cells within *T. gondii*-infected cell layers into S phase which also favored for parasite invasion.

The cell cycle of mammalian cells represents a highly regulated and complex process that includes successive progression of distinct cell cycle phases [G(Gap)_0_-, G_1_-, S (synthesis), G_2_- and mitosis phase] finally leading to cell division via cytokinesis^15,16^. The transition to each phase is tightly regulated by specific checkpoints and is based on sequential activation or inactivation of cyclin-dependent kinases (Cdks) and presence or absence of phase-specific cyclins. After cell division, daughter cells enter into G1 phase during which they produce proteins and organelles needed for DNA synthesis in S phase. After DNA duplication, cells enter into G2 phase and are prepared to progress to mitosis (M phase). M phase is composed of five steps: prophase, metaphase, anaphase, telophase, and cytokinesis^17^. To successfully complete mitosis, several proteins and structures need to be formed with one of them known as mitotic spindles. The mitotic spindle promotes correct localization and migration of chromosomes in all mitosis steps. This process is highly controlled and needs correct formation and localization of centrosomes to guide chromosomes to each cell pole of the cell. Several microtubule proteins are involved not only in mitosis but also in cytokinesis, in which the cytoplasm content is finally divided into two daughter cells. Mitosis and cytokinesis are regulated by cyclins, cyclin-dependent kinases, RhoA and Aurora B amongst others^17–19^.

Given that mainly immortalized or tumor cell lines were used in the past in cell cycle-related studies on *T. gondii* infections, which may not reflect the actual situation within primary cells, and that recent data indicated cell type-specific reactions, we here aimed to analyze the impact of *T. gondii* tachyzoites on host cell cycle progression in primary endothelial cells, i.e. in a cell type that is indeed infected by this parasite stage *in vivo*.

## Results

### Toxoplasma gondii tachyzoite infections trigger endothelial host cell proliferation and karyokinesis in primary endothelial host cells

**Figure 1:**
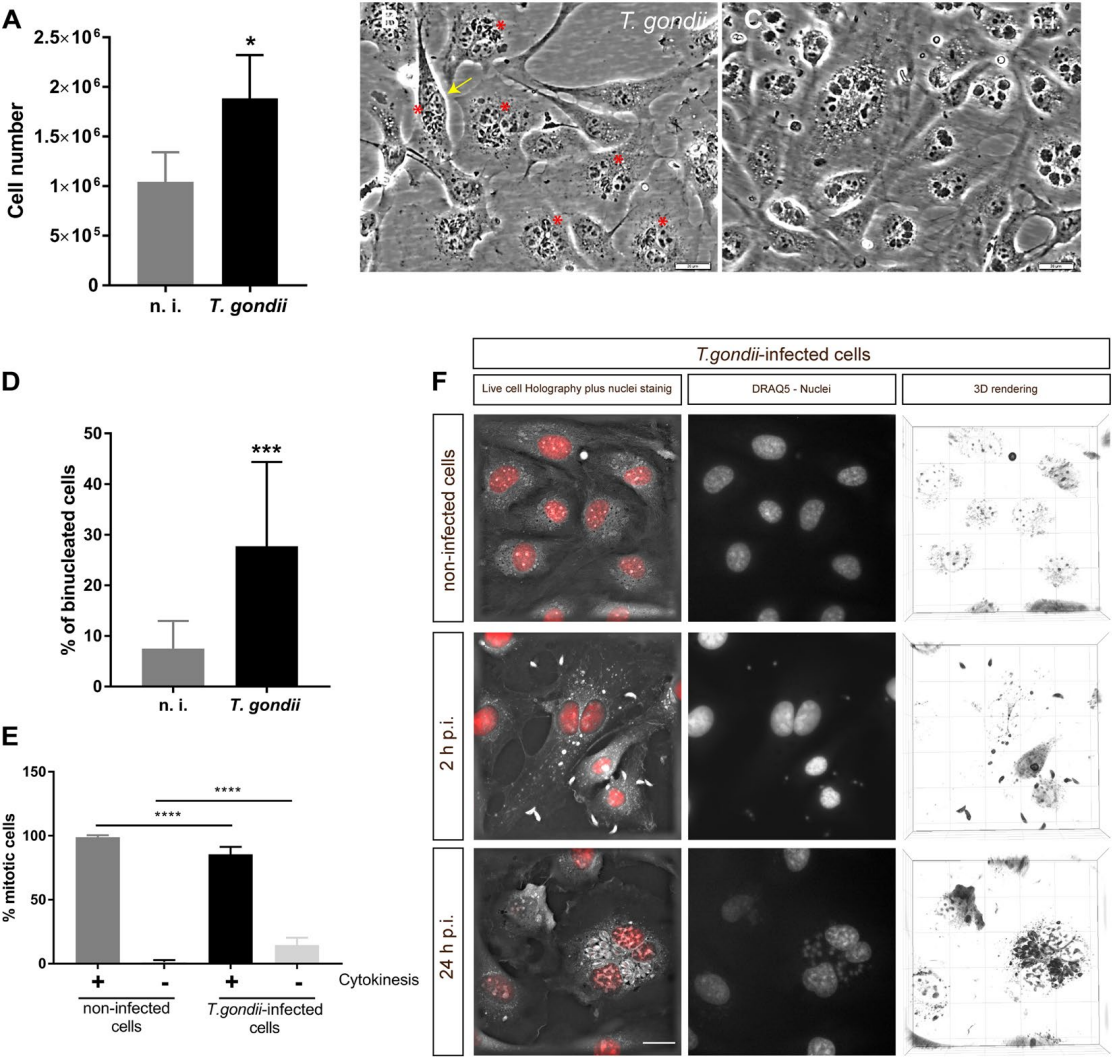
*Toxoplasma gondii*-induced influence on host cell proliferation, karyokinesis and cytokinesis. Sub-confluent primary endothelial cells were infected with *T. gondii* at MOI 5:1 and analyzed after 24 h p.i. (**A**) cell proliferation was estimated by analyzing cell numbers from *T. gondii*-infected BUVEC and non-infected controls (**B**–**D**). The proportion of binucleated cells in *T. gondii*-infected, BUVEC (B, asterisks indicate binucleated cells, the yellow arrow shows a cell in division process) and non-infected controls was estimated by microscopic analysis. (**D**) The number of binucleated cells after DAPI-staining was graphed as percentage of the total cells. (**E**). The proportion of mitotic cells undergoing proper cytokinesis was estimated by time-lapse-based monitoring *T. gondii*-infected and non-infected cells (n > 1000, each) on single cell level for 20 h of recording using phase contrast microscopy. (F) 3D -holotomographic illustration of binucleate *T. gondii*-infected BUVEC at 2 and 24 hours p. i. The nuclei were stained with vital staining probe DRAQ5 (red). Statistical analysis: (A and D) *t-*test or (E) Nonparametric one-way ANOVA (Kruskal-Wallis post-test). *p ≤ 0.05, ***p ≤ 0.001, ****p < 0.0001.

The effect of *T. gondii* infection on host cell proliferation was examined in a simplistic approach by counting BUVEC within an infection kinetics of 6–24 h. Given that we worked with a primary cell type, considerable variations in cell counts per area are common and often conceal significant reactions^20,21^. To give attribute to this phenomenon, we worked with six biological replicates and used identical cell numbers for seeding. Since *T. gondii* tachyzoite infections lead to enhanced host cell lysis from 24 h p. i. onwards in BUVEC, which obviously will falsify cell enumeration, the experiments were restricted to one day p. i. We here achieved an infection rate of 40 ± 10% in BUVEC using an MOI of 5:1. Overall, *T. gondii* infections led to an enhanced host cell proliferation which was already apparent 12 h p. i. (data not shown) but became statistically significant only with 24 h p. i. (infected cells vs. controls: *p* = 0.0181, Fig. 1A).

By applying conventional phase contrast (Fig. 1B,C) and holotomographic microscopy (3D Cell Explorer-fluo, Nanolive, Fig. 1F) combined with a vital staining of nuclei by DRAQ5, we consistently observed that *T. gondii*-infected BUVEC often showed two or more host cell nuclei during *in vitro* development (BUVEC are very flat endothelial cells thereby easily allowing nucleus identification) (Fig. 1B,C,F). For quantification, samples from non-infected and *T. gondii-*infected cells were fixed and stained with DAPI for nuclei detection. Microscopic quantitative analyses showed the number of nuclei ranging from two up to five per host cells, with most host cells being binucleate. Thus, at 24 h p. i., almost a third (27.7%) of *T. gondii*-infected BUVEC had acquired a bi/multinucleate phenotype compared to a proportion of 7.5% in non-infected BUVEC within the same cell layer (infected *vs*. controls: *p* < 0.0016) thereby indicating parasite-triggered host cellular karyokinesis (i.e. nuclear division) (Fig. 1D). To follow the mitotic behavior of binucleate cells and to estimate whether correct cytokinesis indeed occurs in these cells, time-lapse-based recordings of *T. gondii*-infected BUVEC layers were performed over a period of 20 h in a temperature and CO_2_-controlled system using a top-stage-incubator. In total, ~2500 cells were analyzed for cytokinesis in infected and non-infected mitotic cells (Fig. S1A and B-cropped video from the original, to show the cytokinesis failure in *T. gondii-*infected cells). Interestingly, a significantly higher proportion (14.5%; *p* < 0,0001) of *T. gondii*-infected cells failed to successfully accomplish cytokinesis in contrast to a much lower proportion (1.2%) within the non-infected cell population (Fig. 1E and Supplementary video 1A-B). These observations proved that *T. gondii* tachyzoite infections significantly influence host cell cytokinesis.

### Cell cycle arrest of *T. gondii*-infected BUVEC occurs in G2-M phase and is independent of G2-specific cyclin B1 expression

**Figure 2:**
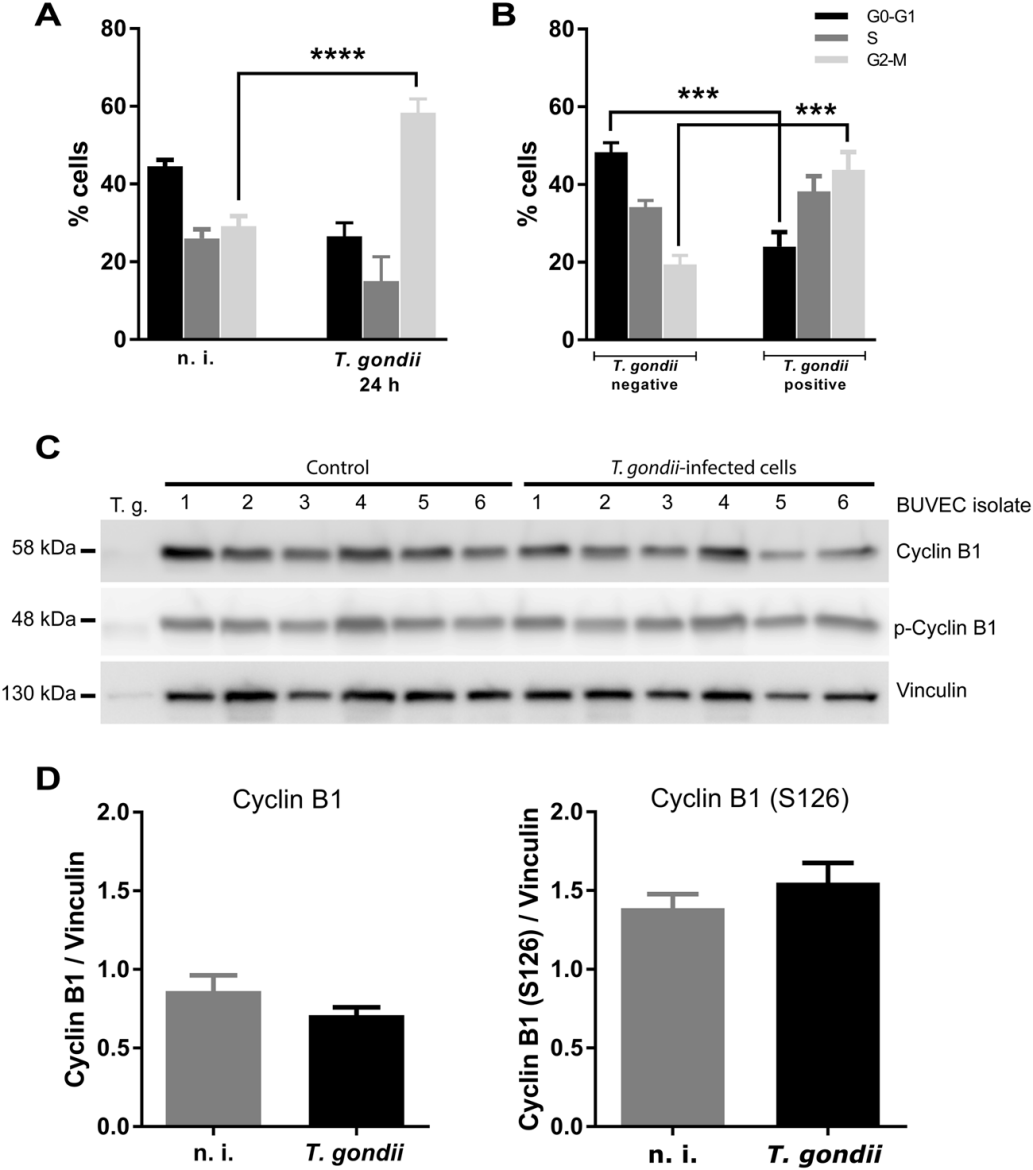
Distribution of cell cycle phases in *T. gondii*-infected BUVEC and control cells and expression/phosphorylation of cyclin B1 in infected cells. (**A**) BUVEC were infected with *T. gondii* tachyzoites and examined for DNA content one day after infection applying FACS analyses. Therefore, the total amount of cells with one (G-phase) or two copies (G2-phase) of the genome was plotted as a percentage of the total cells vs DNA amount. (**B**) Analysis of *T. gondii*-infected cells *vs* non-infected cells originating from the same (infected) cell layer was done using an specific antibody against *T. gondii* tachyzoites for splitting both population. (**C**,**D**) Analysis of cyclin B1 expression and phosphorylation in *T. gondii*-infected BUVEC. Six biological replicates of BUVEC were analyzed by Western blotting (**C**) for the expression of cyclin B1 and its phosphorylated form (S126). For control, pure *T. gondii* tachyzoites were also analyzed. The density of the protein signals was quantified and graphed as ratio relative to vinculin as housekeeping protein (**D**). Bars represent the median ± SEM. Nonparametric one-way ANOVA, ***p ≤ 0.001, ***p < 0.0001.

To estimate whether *T. gondii* infections indeed dysregulate host cellular cell cycle progression in BUVEC, we performed FACS-based analyses on the cellular DNA content (for exemplary gating process, see Fig. S2-A, B). This well-established method allows the discrimination of three main periods of the cell cycle (G0/G1-, S-, G2/M-phase) but cannot distinguish between the single phases G0- and G1- or G2- and M. In a first approach, we compared total cell layer samples of *T. gondii*-infected BUVEC with non-infected controls without discriminating between infected and non-infected cells within the same cell layer in the former samples (for gating process, see Fig. S2A). Here, a significantly higher proportion of cells were present in G2-M-phase in *T. gondii*-infected BUVEC layer samples when compared to control cell layers (*p* = 0.0007, Fig. 2A). For a more precise approach, we analyzed individual *T. gondii*-infected and non-infected cells within the same infected cell layer by additionally staining by a *T. gondii*-specific antibody (gating process in Fig. S2B). In line, these analyses confirmed the data on total cell layers and revealed a significantly increased proportion of *T. gondii*-infected BUVEC in G2-M-phase when compared to non-infected control cells (G2-M-arrest, infected vs. non-infected cells: *p* = 0.0008, Fig. 2B). Simultaneously, a significantly decreased proportion of infected BUVEC were found in G0/G1-phase (infected vs. non-infected cells: *p* = 0.0002) whilst those in S phase remained stable. These data indicated a stasis of *T. gondii*-infected BUVEC either in G2- or in M-phase (or both).

We further tested for a paracrine effect of parasite infection by supplementing non-infected BUVEC with infection-conditioned medium (filtered supernatant of BUVEC infected with *T. gondii* tachyzoites for 24 hours) or with supernatant from non-infected controls. Overall, no paracrine effect of *T. gondii*-conditioned medium on non-infected BUVEC was observed, neither by FACS, nor by cell numbers or by microscopic observation of binucleated cell phenotypes (Fig. S3-A, B).

In addition, we controlled for the abundance of a key molecule of G2-phase, i. e. cyclin B1, in its phosphorylated (at S126) and non-phosphorylated form. Western blot-based analyses on six different BUVEC isolates showed that cyclin B1 was neither altered in its expression nor phosphorylation status in *T. gondii*-infected BUVEC when compared to non-infected control cells (see Fig. 2C,D) thereby indicating that parasite-driven alterations of the host cellular cell cycle may rather be attributed to M- than to G2-phase.

### *T. gondii* tachyzoite infections affect mitosis by propagating host cellular chromosome segregation errors and inducing supernumerary centrosome formation

**Figure 3:**
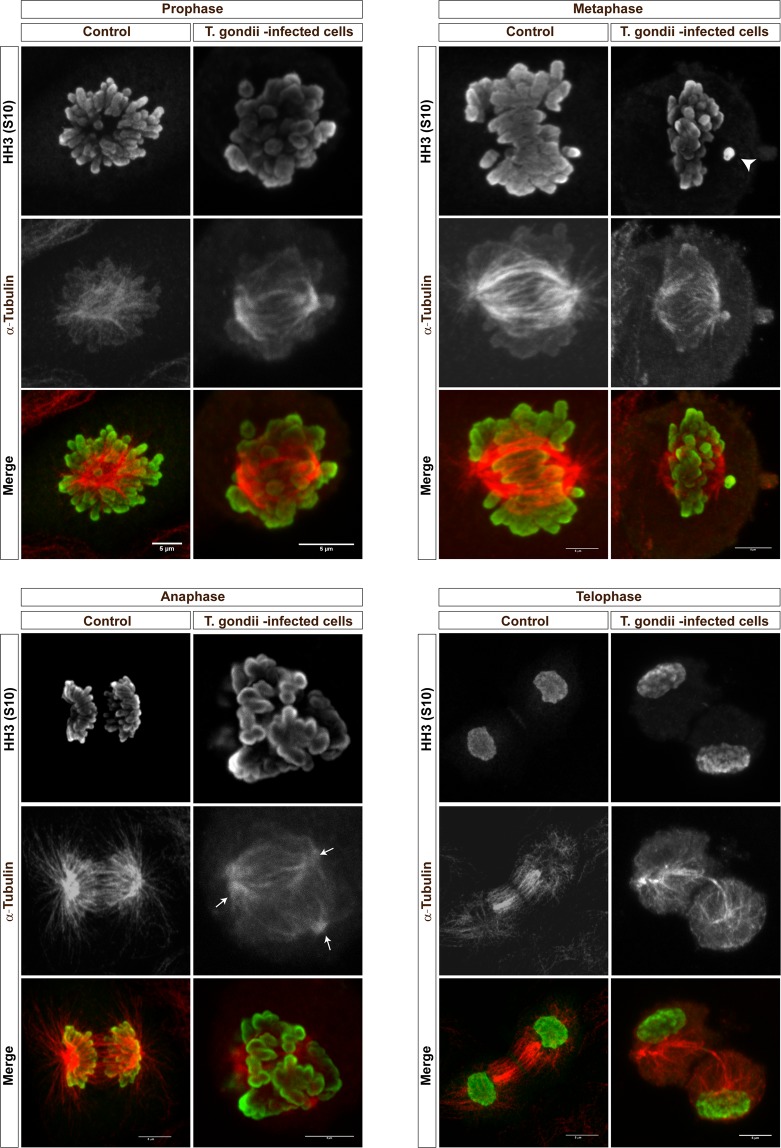
Chromosome segregation of mitotic *T. gondii*-infected BUVEC. Mitotic *T. gondii*-infected BUVEC and non-infected cells were stained for chromosomes via phosphor-HH3 S10 (green) and for mitotic spindle via α-Tubulin (red) and analyzed via confocal microscopy. Arrowhead indicates a disalignment of a chromosome (metaphase), white arrows indicate the presence of more than two-spindle poles in anaphase. Scale bar: 5 µm.

**Figure 4:**
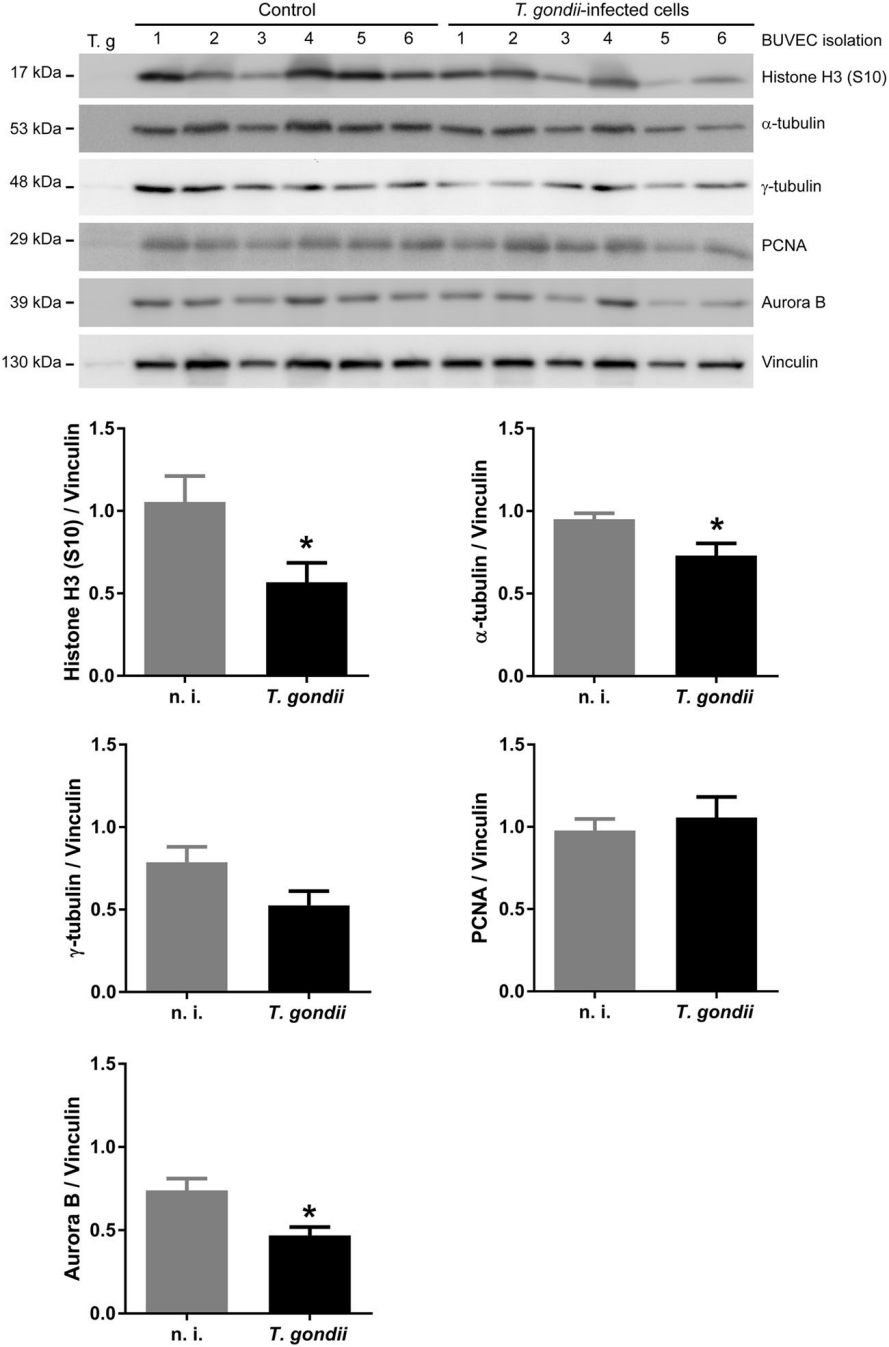
Histone H3, α-tubulin, γ-tubulin, PCNA and Aurora B expression in *T. gondii*-infected BUVEC. Protein extracts from non-infected and *T. gondii-*infected BUVEC (six biological replicates) were subjected to Western blotting and analyzed for Histone H3 (S10), α-tubulin, γ-tubulin, PCNA and Aurora B expression. Pure *T. gondii* tachyzoites were analyzed in parallel for control reasons. The density of the protein signals was quantified and graphed as ratio relative to vinculin as housekeeping protein. Bars represent the median ± SEM. Bars represent the median ± SEM. *t-*test, *p ≤ 0.05.

Given that data on cyclin B1 expression indicated that *T. gondii*-infected BUVEC may not be arrested in G2-phase but rather rapidly be driven into M-phase (which is also in line with an enhanced proportion of infected cells in G2/M-phase), we here performed mitosis-related analyses on host cellular chromosome decondensation and segregation. By using confocal microscopy we applied a double immunostaining for a classical marker of mitosis-related chromosome decondensation via detecting the phosphorylated form of histone H3 ([phospho-histone H3 (S10)], and for the microtubule marker α-tubulin to illustrate chromosome segregation and mitotic spindle formation. Remarkably, at 24 h p. i. a high proportion (aprox. 15%) of mitotic cells showed a severe impairment of chromosome segregation when being infected with *T. gondii* tachyzoites. Alterations varied from chromosomes being displaced out of the equatorial plane to different aberrant shapes leading to dramatic chromosome missegregation (please see Fig. 3). At prophase, an early, but seemingly irregular condensation of microtubules was observed (Fig. 3). During metaphase, the spindles appeared more compact and seemed to lose the defined shape of polar spindles. Interestingly, in this cell cycle phase, we also detected some chromosomes being outside of the mitotic equatorial plane of alignment (Fig. 3, metaphase, white arrow). Chromosome missegregation was spread along anaphase and cells showing more than two mitotic spindle poles were observed (Fig. 3, anaphase, white arrows). Intriguingly, even cells lacking proper chromosome segregation reached telophase, performed nucleokinesis (as visualized by two host cell nuclei, Fig. 3, telophase) and induced reorganization of the tubulin cytoskeleton which is an obligatory step for mitosis (Fig. 3, telophase). To estimate S10 phophorylation of histone H3 and α-tubulin expression in *T. gondii*-infected BUVEC, immunoblot-based analyses were performed. When normalizing to vinculin expression, both molecules were found reduced in their abundance in infected cells (Fig. 4; infected *vs* non-infected cells: histone H3: *p* = 0.0333; α-tubulin: *p* = 0.0228). We additionally attempted to normalize S10-phosphorylated histone H3 expression to total histone H3 abundance but failed to obtain reliable data due to poor Western blot qualities based on sub-optimal antibody binding in the bovine system.

**Figure 5:**
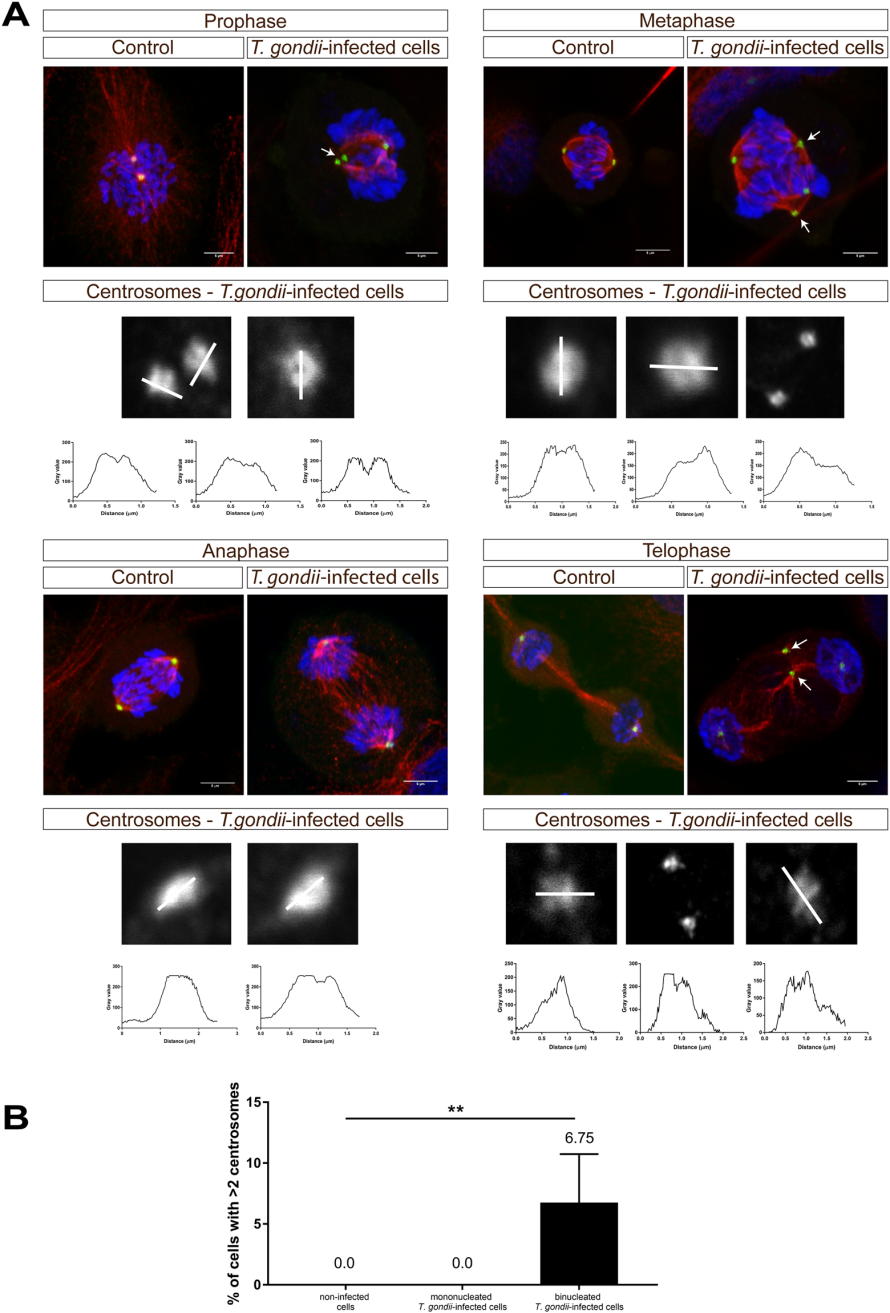
Mitotic spindle and centromer formation in *T. gondii*-infected BUVEC. (**A**) *T. gondii*-infected BUVEC and control cells were stained for chromosomes by DAPI (blue), for mitotic spindles by α-Tubulin (red) and for centromeres by γ-Tubulin (green) and analyzed via confocal microscopy. Additionally, the intensity of centromer-related signals was assessed and plotted as a graph showing intensity value *vs* distance (in µm). Scale bar represents 5 µm. (**B**) The number of centrosomes in mitotic cells, in T.gondii-infected BUVEC and control cells, were counted, and graph as a percentage of the total mitotic cells in the monolayer. Bars represent the median ± SEM. Bars represent the median ± SEM. Nonparametric one-way ANOVA (Kruskal-Wallis post-test), **p ≤ 0.001.

Alterations of chromosome segregation may lead to multipolar mitosis and the presence of additional centrosomes might induce supernumerary spindle pole formation which then impairs proper chromosome distribution and causes chromosome misdirection to three or more poles during anaphase^22,23^. To control for adequate centrosome formation, we performed confocal microscopy-based analyses using γ-tubulin as specific centrosome marker. Here, we indeed detected supernumerary centrosomes especially during pro- and metaphase in *T. gondii*-infected BUVEC (Fig. 5A) indicating the presence of multipolar mitosis. For quantitative analysis, we counted the numbers of centrosomes in mitotic binucleated *T. gondii*-infected cells and in non-infected mitotic control cells. As expected, all mitotic non-infected cells and all mitotic mononucleated *T. gondii*-infected BUVEC showed only two centromeres whilst in mitotic binucleated *T. gondii*-infected cells, the proportion of cells carrying more than two centrosomes was significantly enhanced (*p* = 0.0002, Fig. 5B).

In order to analyze whether the centrosomes of infected BUVEC contained one or two centrioles, the intensity of γ-tubulin-based fluorescence signals was estimated by using Fiji Analyze plot profile plugins and Graph Pad-based plotting. In most cases, the signal showed two spines thereby indicating the presence of two centrioles (except for mainly one peak in telophase) (Fig. 5A). Interestingly, as detected by immunoblotting, γ-tubulin abundance was found reduced in *T. gondii*-infected cells (but not on a significant level, Fig. 4), which may be explained by a reduced mass of supernumerary centrosomes. Overall, our data revealed for the first time that *T. gondii* tachyzoite infections caused aberrant chromosome segregation and impaired mitotic spindle formation in BUVEC thereby massively affecting host cellular mitosis.

### The two nuclei of binucleated *T. gondii*-infected host cells are in a different cell cycle phase

**Figure 6:**
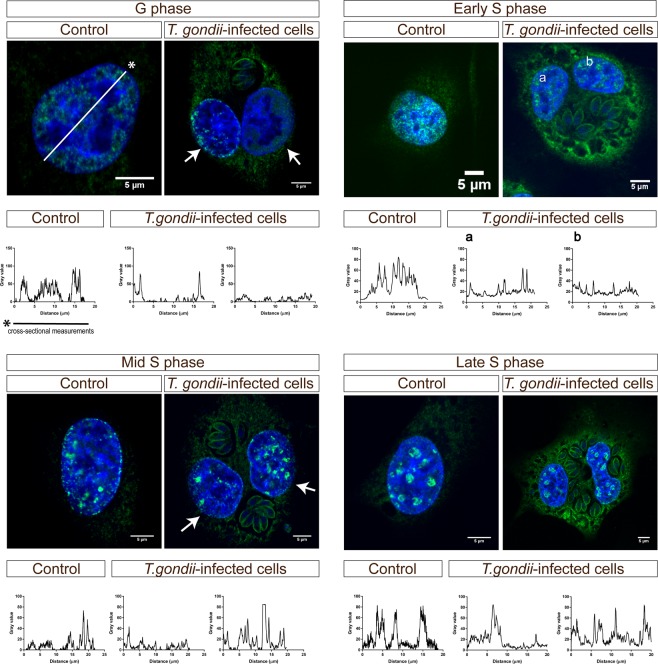
Expression of PCNA in binucleate *T. gondii*-infected BUVEC. *T. gondii*-infected BUVEC and control cells were stained for DNA via DAPI (blue) and for Proliferating cell nuclear antigen (PCNA, green) and analyzed via confocal microscopy. The nuclei of binucleate cells are indicated by white arrows. To analyze the nuclear PCNA distribution, we measured the intensity of PCNA-related signals by drawing a longitudinal line over the nuclei and plotting these data as a graph showing intensity value *vs* distance (in µm). (**a**) = left and (**b**) = right nucleus of a binucleate cell. Scale bar represents 5 µm.

The precise pattern of proliferating cell nuclear antigen (PCNA) within the nucleus during different cell cycle phases was initially described by Bravo *et al*., in 1985. PCNA nuclear distribution is characteristic for G phase, early-, mid- and late-S phase and is therefore generally utilized as a marker for sub-stages of DNA replication^24^. One goal of this experiment was to examine whether a certain proportion of BUVEC may show different phases of S-phase since this could have helped to elucidate whether cell proliferation (Fig. 1A) would origin from infected or non-infected cells within the monolayer. In addition, the initial damage leading to mitosis impairment in *T. gondii*-infected host cells as described above might already have occurred in the preceding S phase. Thus, we here performed analyses on S phase progression using a PCNA-based approach. Using immunostaining, we here detected PCNA distribution in nuclei of *T. gondii*-infected BUVEC at 24 h p. i. and compared it to non-infected control cells (Fig. 6). As an interesting finding, we observed that the two nuclei of single *T. gondii*-infected BUVECs consistently revealed different S-phase states. Thus, some *T. gondii*-infected cells contained one nucleus in G phase whilst the others passed through mid-S-phase (Fig. 6, G phase, indicated by arrows). Similarly, we found cells displaying one nucleus in mid- and others in late-S phase (Fig. 6, mid-S phase, indicated by arrows). In line, cross-sectional intensity measurements of PCNA-derived fluorescence signals performed on single nucleus level confirmed a differential PCNA distribution in the two nuclei of single *T. gondii-*infected BUVEC and in comparison to non-infected control cells (Fig. 6). Important to note, infected cells also showed PCNA signals at tachyzoite surfaces and throughout the host cellular cytoplasm (Fig. 6). However, immunoblotting-based quantification of PCNA expression did not reveal significant differences in PCNA abundance between *T. gondii*- and non-infected cells (Fig. 4). As such, the detected differences may not account to alterations in cellular protein abundance but to a change in subcellular distribution.

### *T. gondii* infection impairs host cell cytokinesis-related Aurora B expression

**Figure 7:**
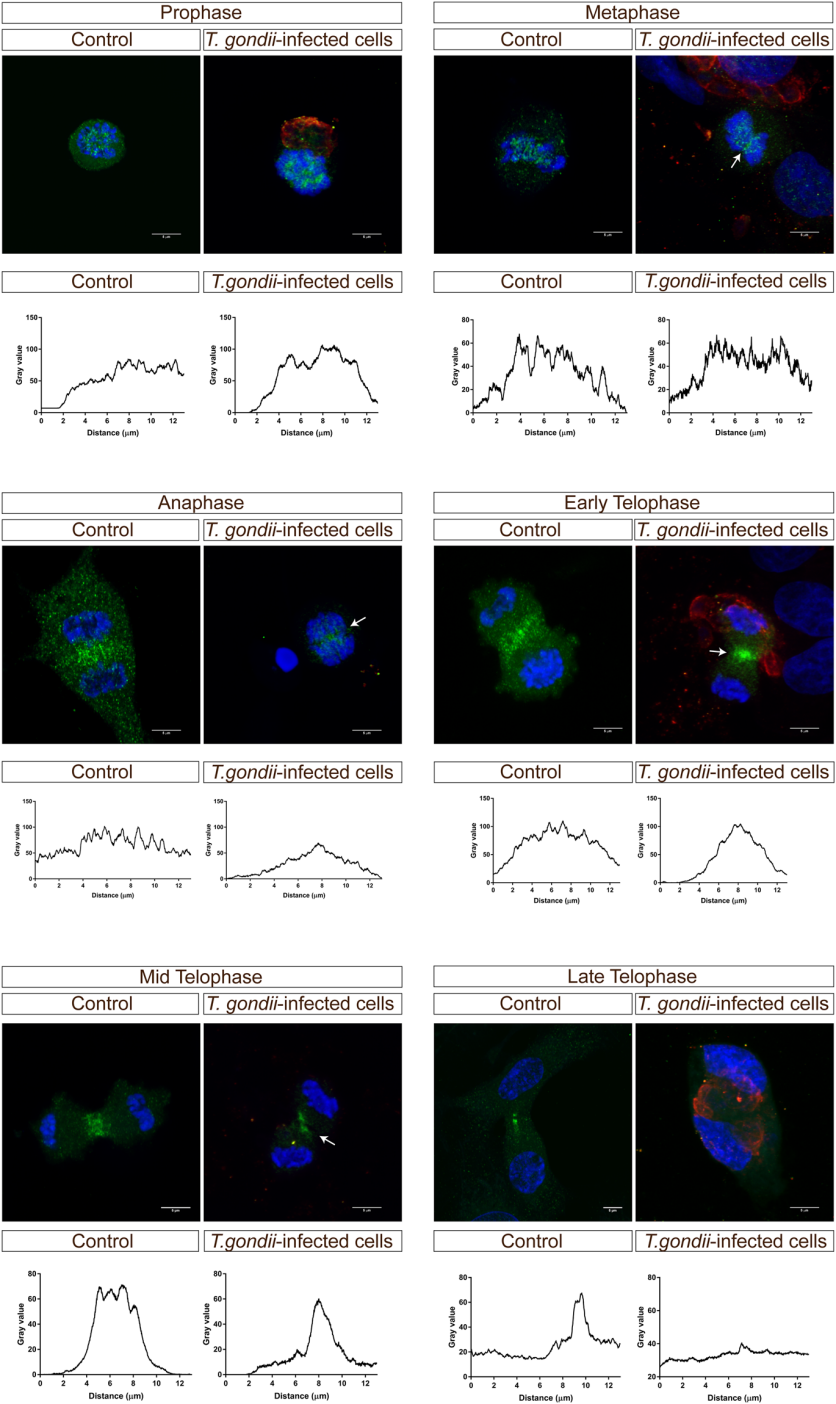
Aurora B expression in *T. gondii*–infected BUVEC. Mitotic *T. gondii*-infected BUVEC and control cells were stained for DNA via DAPI (blue), for T. gondii tachyzoites (red) and for Aurora B (green) and analyzed via confocal microscopy. To analyze the extension of Aurora B expression within the cell, we measured the intensity of Aurora B-related signals along a 13 µm-line and plotted these data as a graph showing intensity value *vs* distance (in µm). White arrows indicate Aurora B expression in *T*. gondii-infected cells. Scale bar represents 5 µm.

According to the data presented above (Fig. 1C), a significantly enhanced cytokinesis failure was observed in *T. gondii*-infected cells. Cytokinesis is the final step of cell division and it begins during chromosome segregation (reviewed in^25^). Cytokinesis can be divided into four stages: cleavage plane, ingression of the cleavage furrow, formation of the midbody and abscission. Aurora B kinase is involved in regulating the cleavage of polar spindle microtubules and represents a key regulator of the onset of cytokinesis. We therefore analyzed Aurora B abundance via immunoblotting and found significantly reduced levels of this molecule in *T. gondii*-infected cells (infected vs. controls: *p* = 0.0157, Fig. 4) indicating that Aurora B dysregulation may be involved in *T. gondii*-triggered cytokinesis impairment. In line, confocal microscopy revealed an altered distribution of this molecule in *T. gondii*-infected BUVEC (Fig. 7). Even though we were able to detect Aurora B expression in all stages of mitosis and it was indeed localized in the cleavage furrow (Fig. 7- mid telophase), measurements of Aurora B signals showed comparable intensities but decreased expansion in dividing cells (8 µm in *T. gondii*-infected cells *vs*. 13 µm in control cells), especially from pro- to meta/early telophase. Furthermore, in contrast to control cells, Aurora B was no longer detectable in late telophase of *T. gondii*-infected BUVEC.

## Discussion

*T. gondii* is well-known for its highly sophisticated capacities to modulate its host cell for successful intracellular development and proliferation. Amongst diverse other functional categories, earlier studies showed that *T. gondii* infections dampen host cell proliferation and lead to host cell cycle arrest^12^. The fact that *T. gondii*-triggered cell cycle blockage was attributed to different cell cycle phases in different host cell types^12,14^ might indicate cell type-related reactions. In line, in the current study, we show that in primary host endothelial cells, *T. gondii* does not dampen but induces host cell proliferation and at the same time drives primary host endothelial cells into G2-M-phase. For the first time, we here show that *T. gondii*-driven impairment of G2-M-phase mainly concerned the mitotic phase of host cells by propagating chromosome segregation errors, mitotic spindle alteration, the formation of supernumerary centrosomes and cytokinesis failure in primary endothelial cells. Interestingly, some of these phenomena were already described in a human fibroblast cell line, where the authors show that *T. gondii-*infected cells commonly fail to finish cytokinesis resulting in larger, multinucleated cells^26^.

In the current study, we analyzed the influence of *T. gondii* on primary BUVEC proliferation. We chose this primary cell type to be rather close to the *in vivo* situation and to avoid false influences on cell cycle or division activities of the host cell driven by cell immortalization or tumoral origin^27^. In contrast to data on other cell types^12,28^, BUVEC-related analyses showed that *T. gondii* infections induced an increase of host cell proliferation. Important to note, we here exclusively analyzed host cell layers for up to 24 h p. i. to avoid false negative influence driven by parasite-induced host cell lysis. This contrasts to experimental conditions of other previous studies in which host cell proliferation for up to 48 h p. i.^12^ or even 120 h p. i.^28^ was analyzed. However, current findings may still reflect cell type-specific reactions as already reported for other parasite-infected host cells^29^.

Besides reducing host cell proliferation, *T. gondii* was reported to arrest host cell cycle before progression to mitosis^12,14,28^. This finding could not be verified in the BUVEC-based infection system. Thus, as an incidental microscopic finding, we consistently observed that *T. gondii*-infected BUVEC were indeed mitotically active and - in contrast to neighboring non-infected cells - showed a multinucleate phenotype that indicated nuclear division (karyokinesis). Correspondingly, a high percentage of infected BUVEC (27.7%) had acquired a bi/multinucleate phenotype at 24 h p. i. in contrast to only 6.8% binuclearity in non-infected controls which indicated that infected BUVEC had at least entered mitosis. In line, a binucleated phenotype was also described for *Plasmodium* spp.- and *T. cruzi*-infected host cells and authors attributed this phenomenon to cytokinesis impairment^29,30^. In accordance, we could here demonstrate that a significant proportion of *T. gondii*-infected BUVEC experienced cytokinesis failure. Interestingly, in case of *T. cruzi*-infected cells, the proportion of multinucleate cells was similar to that observed here and this phenomenon was linked to large schizont development^29^. Even more interesting, the *T. gondii*-driven formation of multinucleate BUVEC proved as species-specific since enhanced karyokinesis could neither be observed in BUVEC infected with the closely related parasite species *Besnoitia besnoiti* nor *Neospora caninum* (Z.D. Velasquez, unpublished data). Overall, enhanced karyokinesis contrasts with findings of Molestina *et al*.^14^ und Brunet *et al*.^12^, which described a stasis of *T. gondii*-infected cells in S or G_2_ phase which is obviously causing absence of mitosis.

Analyses on the cellular DNA content confirmed that *T. gondii* indeed dysregulates host cellular cell cycle progression in BUVEC by increasing cell stasis in G2-M-phase and by simultaneously lowering the proportion of BUVEC in G0-G1-phase. It has to be noted that by the here applied method of DNA content determination it is not possible to discriminate between the single G2- and M-phases. Given that the abundance or phosphorylation of G2-specific cyclin B1 was not affected in *T. gondii*-infected BUVEC, G2-phase did not seem to be the major target of cell cycle dysregulation in this cell type. These data partially agree with earlier findings reporting on an infection-driven host cellular arrest in G2-phase^12^ and contrasts to reports on a shift from G0/G1 to S-phase^13,14^ or even both^28^. Moreover, stasis of *T. gondii*-infected human foreskin fibroblasts during S-phase at G2/M border was accompanied by a delayed or absent increase of cyclins A and B in combination with an early elevation of cyclin E_1_ levels^14^. In contrast, *T. gondii*-triggered G2-arrest in human dermal fibroblasts or in a human trophoblast cell line was supported by a diminished abundance of cyclin B_1_ whilst G2/M checkpoint-related molecules were not affected^12^. Overall, these data indicate that *T. gondii*-triggered cell cycle-related impairment is host cell type-dependent and may therefore especially differ between primary and permanent cell lines. The most obvious argument for this assumption comes from data on *Plasmodium* infections showing that a parasite-driven impairment of the hepatocyte host cell cycle could only be demonstrated in *Plasmodium*-infected HepG2 cells^30^ whilst it did not play a role in primary hepatocytes (which are generally quiescent) or in *in vivo* mouse models. Furthermore, it is important to consider the *T. gondii* strain that was used in this study as a factor in the contradict results previously reported in the literature. Here, we have been worked with a RH strain which is a highly virulent culture-adapted type I strain that undergoes extremely rapid proliferation^31,32^.

The current data show for the first time that *T. gondii* tachyzoite infection impairs chromosome segregation of host cells. Thus, mitotic *T. gondii-*infected BUVEC revealed chromosome misalignment in addition to chromosomes migrating to more than two poles. The process of chromosome segregation is highly regulated and requires numerous factors for adequate processing, such as molecules being involved in DNA movement, in linkage of DNA to cellular structures and in chromosome maintenance^33^. Tubulins are essential for proper chromosome-cell-linkage and they form part of the mitotic spindle structure, which is fundamental for chromosome segregation^34–36^. In the onset of mitosis, in a process termed centrosome maturation, the γ-tubulin ring complex is attached to mitotic centrosomes activating the mitosis process^37^. In a physiological setting, centrosomes are doubled during cell cycle and the presence of one centrosome at each cell pole ensures the correct localization of the mitotic spindle. We here demonstrate that *T. gondii-*infected BUVEC beared a lower α-tubulin abundance than control cells and that the shape of mitotic spindles was structurally altered already in prophase and more dramatically in anaphase leading to aberrant chromosome segregation and cell spindle formation. In addition, *T. gondii* tachyzoite infection induced the formation of supernumerary centrosomes which may obviously cause improper multipolar mitosis, such as tripolar mitosis^38^. Multipolar spindles are often associated with supernumerary centrosomes and chromosomal instability^37,39,40^ and may result from the activation of oncogenic kinases that control centrosome duplication and/or the loss of tumor-suppressor genes^41–43^. Multipolar mitosis is commonly described to occur in malignant lesions and has been suspected to contribute to oncogenesis for over a century^44–46^. Given that multipolaricity may also be based on excess centriole formation, we analyzed the intensity peaks of single centrosomes and found that centriole formation was not significantly altered in *T. gondii*-infected BUVEC. Earlier reports indicated that supernumerary centrosomes may be transient and do not necessarily lead to the formation of (>2) aneuploid daughter cells^47,48^. In line, we observed via life cell imaging that most binucleate *T. gondii*-infected BUVEC either fail to divide before parasite-driven cell lysis or indeed form only two daughter cells. Also, it is possible to explain this phenomenom by considering cytoskeleton remodelation when the parasites enter into the host-cells. In this context, Walker *et al*.^26^, demonstrated infections with *T. gondii* tachyzoite induce a remodelation of the host cell tubulin cytoskeleton, controlling α-tubulin abundance around the *T. gondii* parasitophorous vacuole membrane (PVM)^26^. If *T. gondii* scavenges host cellular tubulin cytoskeleton for PVM formation, the host cell in turn would lack parts of this protein for proper mitotic spindle formation.

The structural connection between centrioles is established during S phase and persists until late mitosis and G1-phase^49^. Therefore and to analyze whether BUVEC may also show enhanced S-phase and thereby contribute to enhanced cell proliferation, we also analyzed S-phase progression in *T. gondii*-infected BUVEC by PCNA-related experiments. PCNA is used as a marker of S-phase substages, is critically involved in DNA replication and repair and has cell cycle-dependent properties. Interestingly, it was also described to be involved in both, G1 and G2 arrest in association with p21^50^. As an interesting finding, we showed that the two nuclei of a binucleate *T. gondii*-infected host cell were not necessarily experiencing the same cell cycle phase or substage of S-phase. However, even though we detected with 27.7% a rather large proportion of binucleate cells in *T. gondii*-infected cell layers, the total expression of PCNA did not differ from non-infected control cells. These data may indicate that PCNA was only changed in its distribution but not in its abundance and that binucleate infected cells contain two virtually separate nuclear compartments which experience an individual cell cycle progress thereby eventually compensating different PCNA abundances within one cell.

Cytokinesis represents the final step of the cell division process during which the cytoplasm of the cell is divided into two daughter cells. It is a highly controlled process and impairment of any step in the cascade may result in cytokinesis failure^51–53^. In case of mammalian somatic cells, a cytokinesis failure represents a rare event in culture and occurs even less *in vivo*^54^. Given that the multinucleate phenotype of *T. gondii*-infected BUVEC indicated an impairment of cytokinesis, we here analyzed a key molecule of cytokinesis, Aurora B. Aurora B kinase complex positively regulates cytokinesis and is also involved in chromosome segregation and in a spindle-related checkpoint^55^. Aurora B is a serine-threonine kinase belonging to a highly conserved Aurora family of mitotic kinases which also play a role in tumor genesis^53^. We here show that *T. gondii* induces a downregulation of Aurora B expression and additionally alters its intracellular distribution in infected BUVEC, all of which indicated that proper cytokinesis is impaired in *T. gondii*-infected cells. This assumption was finally proven by live cell imaging experiments in which non-infected and infected cells were monitored for proper cell division over 20 h of recording. These experiments clearly indicated that a significantly enhanced proportion of *T. gondii*-infected BUVEC with multinucleate phenotype did not divide properly before parasite-driven lysis occurred. Consequently, *T. gondii* infection indeed triggers cytokinesis failure in primary endothelial cells. Cytokinesis failure was also assumed to occur in *T. cruzi*- and *Plasmodium* spp.-infected host cells which also presented a binucleated phenotype^29,30^. However, the molecular basis of this phenomenon is unknown and needs to be investigated in future experiments.

So far, it remains unclear how the findings on enhanced BUVEC proliferation fit to cytokinesis impairment. However, given that the infection rate within a cell layer was not 100%, the current data on cell proliferation does not discriminate between the proliferative activities of infected and non-infected cells within the tested cell layer. As such, in principle, increased cell proliferative activity may also be attributed to non-infected cells being stimulated by paracrine effects. Interestingly, Lavine *et al*.^13^ described an enhanced progression of non-infected cells within a *T. gondii*-infected cell layer into S phase. Lavine *et al*. could mimic this effect by parasite-conditioned medium proposing paracrine effects or reactions driven by soluble factors. However, in the current study, a paracrine effect of infection-conditioned medium on non-infected cells could not be stated thereby denying non-infected cells as a source of enhanced proliferation. In this context, it has to be considered that the infection rate was ~40%, but only 15% of all cells present within the monolayer failed to perform cytokinesis. Thus, a considerable proportion of infected cells may have contributed of total cell proliferation. As a hypothesis, it may also be possible that these cells grow faster than non-infected ones and thereby cause an overall increase in cell numbers.

Overall, we here describe for the first time that *T. gondii* infections profoundly affect the cell cycle progression of primary bovine endothelial host cells by propagating several functional alterations, such as chromosome segregation errors, mitotic spindle alteration, and cytokinesis failure. The current results add new data on the topic of parasite-related host cell modulation but also strengthen the assumption that *T. gondii*-mediated cell cycle modulation is cell type-dependent.

## Material and Methods

### Primary bovine umbilical vein endothelial cell isolation and maintenance

Primary bovine umbilical vein endothelial cells (BUVEC) were isolated from umbilical veins obtained from calves born by *sectio caesarea* at the Justus Liebig University Giessen. Therefore, umbilical cords were kept at 4 °C in 0.9% HBSS–HEPES buffer (pH 7.4; Gibco, Grand Island, NY, USA) supplemented with 1% penicillin (500 U/ml; Sigma, St. Louis, MO, USA) and streptomycin (500 μg/ml; Sigma) for a maximum of 16 h before use. For the isolation of endothelial cells, 0.025% collagenase type II (Worthington Biochemical Corporation) suspended in Pucks solution (Gibco) was infused into the lumen of ligated umbilical veins and incubated for 20 min at 37 °C in 5% CO_2_ atmosphere. After gently massaging the umbilical veins, the cell suspension was collected in cell culture medium and supplemented with 1 ml fetal calf serum (FCS, Gibco) in order to inactivate collagenase. After two washes (350 × g, 12 min, 20 °C), cells were resuspended in complete endothelial cell growth medium (ECGM, PromoCell, supplemented with 10% FCS), plated in 25 cm^2^ tissue plastic culture flasks (Greiner) and kept at 37 °C in 5% CO_2_ atmosphere. BUVEC were cultured in modified ECGM medium [EGCM, diluted at 30% in M199 medium, supplemented with 5% FCS (Greiner) and 1% penicillin and streptomycin] with medium changes every 2–3 days. BUVEC cell layers were used for infection after 3 passages *in vitro*. All experiments on bovine primary endothelial cells and parasites were conducted in accordance with the permission of the Institute of Parasitology to work with biological agents up to risk class S3** [allowance according §16 BiostoffVO, Az. GI 000056837, approved by the regional commission of Giessen (Regierungspräsidium Gießen)]. Institutional Ethics Commission of Justus Liebig Universität of Gießen (Germany), and in accordance with the current European Animal Welfare Legislation: ART13TFEU.

### *Toxoplasma gondii* tachyzoite maintenance

*Toxoplasma gondii* (RH strain) tachyzoites were maintained by serial passages in MARC-145 (Meat Animal Research Center-145) layers (the infection rate in MARC was 40–50% in all experiments). Therefore, free-released *T. gondii* tachyzoites were harvested from MARC supernatants, pelleted (400 × *g*, 12 min), counted in a Neubauer chamber, suspended in modECGM and used for sub-confluent BUVEC infections (immunofluorescence assays: 12-well formats; immunoblotting and FACS assays: T-25 flask format). All experiments were performed at an MOI of 1:5 (cells: parasites).

### Protein extraction

Proteins from infected and non-infected BUVEC were extracted by cell sonication (20 s, 5 times) in RIPA buffer (50 mM Tris-HCl, pH 7.4; 1% NP-40; 0.5% Na-deoxycholate; 0.1% SDS; 150 mM NaCl; 2 mM EDTA; 50 mM NaF, all Roth) supplemented with a protease inhibitor cocktail (Sigma-Aldrich). Cell homogenates were centrifuged (10,000 × *g*, 10 min, 4 °C) to sediment intact cells and nuclei. The RIPA buffer-soluble protein content in cell supernatant was quantified via Coomassie Plus (Bradford) Assay Kit (Thermo Scientific) following the manufacturer’s instructions.

### SDS-PAGE and immunoblotting

For immunoblotting, samples were supplemented with 6 M urea. After boiling (95 °C) for 5 min, total proteins (60 µg/slot) were separated in 12% or 15% polyacrylamide gels via electrophoresis (100 V, 1.5 h; *tetra* system, BioRad). Proteins were then transferred to polyvinylidene difluoride (PVDF) membranes (Millipore) (300 mA, 2 h). Blots were blocked in 3% BSA in TBS [50 mM Tris-Cl, pH 7.6; 150 mM NaCl containing 0.1% Tween (blocking solution); Sigma-Aldrich] for 1 h at RT and then incubated in primary antibodies (see Table 1) diluted in blocking solution (overnight, 4 °C). Detection of vinculin was used as loading control for normalization of samples. Following three washings in TBS-Tween 0,1% buffer, blots were incubated in adequate secondary antibody (see Table 1) solutions (diluted in blocking solution, 30 min, RT). Following three further washings in TBS-Tween 0.1% buffer, signal detection was accomplished by an enhanced chemiluminescence detection system (ECL^®^ plus kit, GE Healthcare) and recorded using a ChemoCam Imager (Intas Science Imaging). Protein sizes were controlled by a protein ladder (PageRuler Plus^®^ Prestained Protein Ladder ~10–250 kDa, Thermo Fisher Scientific). Protein band intensity quantification was analyzed using Fiji Gel Analyzer^®^ plugin.

**Table 1:** Primary and secondary antibodies used for immunoblotting.

Antigen	Company	Cat. number	Origin	Dilution
**Primary antibodies**				
Vinculin	Santa Cruz	sc-73614	Mouse	1:1000
Cyclin B1	Abcam	ab32053	Rabbit	1:3000
cyclin B1 Ser126	Abcam	ab133439	Goat	1:1000
Histone H3 S10	Abcam	ab5176	Rabbit	1:1000
α-Tubulin	ThermoFisher	A11126	Mouse	1:3000
γ-Tubulin	Abcam	ab179503	Rabbit	1:2000
Aurora B	Abcam	ab3609	Mouse	1:1000
PCNA	Abcam	ab18197	Rabbit	1:1000
**Secondary antibodies**				
Goat anti-mouse IgG Peroxidase conjugated	Pierce	31430	Mouse	1:40000
Goat anti-rabbit IgG Peroxidase conjugated	Pierce	31460	Rabbit	1:40000

### Immunofluorescence assays

Cell layers were fixed with paraformaldehyde (4% 15 min, RT), washed thrice with PBS and incubated in blocking/permeabilization solution (PBS with 3% BSA, 0.1% saponin; 1 h, RT). Thereafter, samples were incubated in primary antibodies (see Table 2) diluted in blocking/permeabilization solution (overnight, 4 °C, in a humidified chamber). After three washings in PBS, samples were submitted to secondary antibody solution (see Table 2; 30 min, RT, darkness). Cell nuclei were labeled with 4′,6-diamidin-2-phenylindol (DAPI) being present in mounting medium solution (Fluoromount G, ThermoFisher, 495952).

**Table 2:** Primary and secondary antibodies used for immunofluorescence analyses.

Antigen	Company	Cat. number	Origin	Dilution
**Primary antibodies**				
Histone H3 S10	Abcam	ab5176	Rabbit	1:100
α-Tubulin	ThermoFisher	A11126	Mouse	1:100
γ-Tubulin	Abcam	ab179503	Rabbit	1:100
*Toxoplasma gondii*	ThermoFisher	PA1-7256	Goat	1:100
Aurora B	Abcam	ab3609	Mouse	1:100
PCNA	Abcam	ab18197	Rabbit	1:100
**Secondary antibodies**				
AlexaFluor 488	ThermoFisher	A11008	Rabbit	1:500
AlexaFluor 488	ThermoFisher	A11001	Mouse	1:500
AlexaFluor 594	ThermoFisher	R37117	Rabbit	1:500
AlexaFluor 594	ThermoFisher	A11005	Mouse	1:500

### Quantification of binucleated cells

BUVEC (three biological replicates) were infected with *T. gondii* for 24 h, fixed with PFA (4%) for 15 min at RT and afterwards stained by DAPI for nuclei detection. Then, the numbers of mono- and binucleated cells within *T. gondii*-infected cell layers or non-infected controls were analysed. All values were presented as a percentage of total number of cells.

### Flow cytometry-based analysis of cell cycle phases

Cellular DNA content was measured using the FxCycle Far^®^ red stain reagent (Invitrogen, F10348) according to the manufacturer’s instructions. Fixation was performed by using BD Fixation/Permeabilization Solution Kit (554714, BD Bioscience). Two experimental approaches were done: first, to determine the general effect of *T. gondii* infection on the host cell cycle we directly analyzed the DNA content in non-infected control and *T. gondii*-infected cell layers without dissecting between infected and non-infected cells in the latter sample. Second, infected monolayers were stained by a specific *T. gondii* antibody and cell populations were discriminated into parasite-positive or -negative (for exemplary gating process, see Fig. S2B). In all cases, the samples were analyzed by a FACSCalibur^®^ Analyzer (Becton-Dickinson, Heidelberg, Germany) applying 633/5 nm excitation and emission collected in a 660/20 band pass. Cells were gated according to their size and granularity. Exclusively morphologically intact cells were included in the analysis. Data analysis was performed by the use of FlowJo^®^ (version 10.5.0) flow cytometry analysis software (FlowJo LLC, Ashland, OR).

To estimate a potential infection-driven paracrine effect, we incubated non-infected BUVEC isolates (N = 4) with filtered (0.2 μm filter) supernatants from non-infected BUVEC from cells that had been infected with *T. gondii* for 24 h (Fig. S3-A). After 24 hours of supplementation, the cells were collected, fixed and analyzed by FACS reading for the analysis of the cells cycle phases (FxCycle Far red).

### Confocal microscopy

All immunofluorescence analyses were performed using confocal microscopy (63x magnification with a numerical aperture of 1.4, LSM 710, Olympus). Two types of image acquisition were used: (*i*) multi-channel images which were merged afterward to define the co-localization of the signal, (*ii*) Z-stacks of 0.3–0.5 microns for cell spindle and chromosome detection. Image processing was carried out by Fiji ImageJ^®^ using Z-projection and merged-channel-plugins being restricted to overall adjustment of brightness and contrast.

### Centrosome quantification

BUVEC (three biological replicates) were infected with *T. gondii* for 24 h and afterwards immunostained for centrosomes via γ-tubulin and for chromosomes by phospho-histone 3 (H3; S10) detection. As such, a total of 479 mitotic cells was analysed for the number of centrosomes being present in a single cell. Overall, two distinct mitotic cell populations were considered: (*i*) cells with two nuclei or (*ii*) cell with more than two nuclei. For both cell populations we distinguished between cells with two centrosomes (physiological condition) and cells showing more than two centrosomes (abnormal condition). All values were presented as a percentage of total number of cells.

### Analysis of cytokinesis by time-lapse-based microscopy

BUVEC (three biological replicates) were cultured in 2-well µ-slide chambers (IBIDI^®^, Martinsried, Germany) for 80% of confluence. Plates were mounted on the stage of a motorized inverted microscope (Olympus Microscope IX81) combined with a top-stage incubator (IBIDI^®^, Martinsried, Germany) to control the temperature, humidity and CO_2_ environment. Ten different coordinates, one per each BUVEC isolate, were recorded for 21 h beginning three hours after *T. gondii* infection. At the same time, non-infected and *T. gondii-*infected cells were registered to detect differences associated to the BUVEC isolation and not to the infection *per se*. In total, 10 videos of 144 frames per each condition and BUVEC isolates were analysed. In the videos analysis, 1032 cells were counted for mitosis with consecutive presence or absence of cytokinesis thereby considering both, non-infected and infected cells within the *T. gondii*-infected cell layer.

### Live cell holotomographic microscopy

Holotomographic images were obtained by using 3D Cell Explorer-fluo (Nanolive) microscope equipped with an 60x magnification (λ = 520 nm, sample exposure 0.2 mW/mm^2^) and a depth of field of 30 µm. For host cell nuclei visualization, BUVEC were stained with the vital dye DRAQ5 (DRAQ5™ Fluorescent Probe Solution, ThermoFischer). Images were analyzed using STEVE^®^ software (Nanolive) to obtain a refractive index-based z-stack^56^ and digital staining was applied according to the refractive index of intracellular structures.

### Statistical analysis

The data were always expressed as mean ± SD from six independent experiments. For cell number- and FACS-based assays, one-way analysis of variance (nonparametric ANOVA) with Kruskal-Wallis post-test was performed using GraphPad Prism^®^ 7 software applying a significance level of 5%. For immunoblot-based analyses, unpaired two-tailed T-tests were performed comparing controls *vs* infected cells, with a 95% confidence interval.

## Supplementary information

Supplementary video 1A

Supplementary video 1B

Supplementary figures
