# Supplementary material for: Toxoplasma gondii-induced host cellular cell cycle dysregulation is linked to chromosome missegregation and cytokinesis failure in primary endothelial host cells
Source: Sci Rep. 2019 Aug 29;9:12496. doi: 10.1038/s41598-019-48961-0 (PMC6715697; doi:10.1038/s41598-019-48961-0)
Supplement: Supplementary file 3 — Supplementary figures [file 41598_2019_48961_MOESM3_ESM.pdf]

Toxoplasma gondii-induced host cellular cell cycle dysregulation is linked to chromosome missegregation and cytokinesis failure in primary endothelial host cells

Zahady D. Velasquez[1]*, Ivan Conejeros[1], Camilo Larrazabal[1], Katharina Kerner[2], Carlos Hermosilla[1], Anja Taubert[1]

1 Institute of Parasitology, Biomedical Research Center Seltersberg, Justus Liebig University Giessen, Giessen, Germany.
2 Institute for Hygiene and Infectious Diseases of Animals, Justus-Liebig-University, Giessen, Germany

Supplementary Figures

Fig. S1A

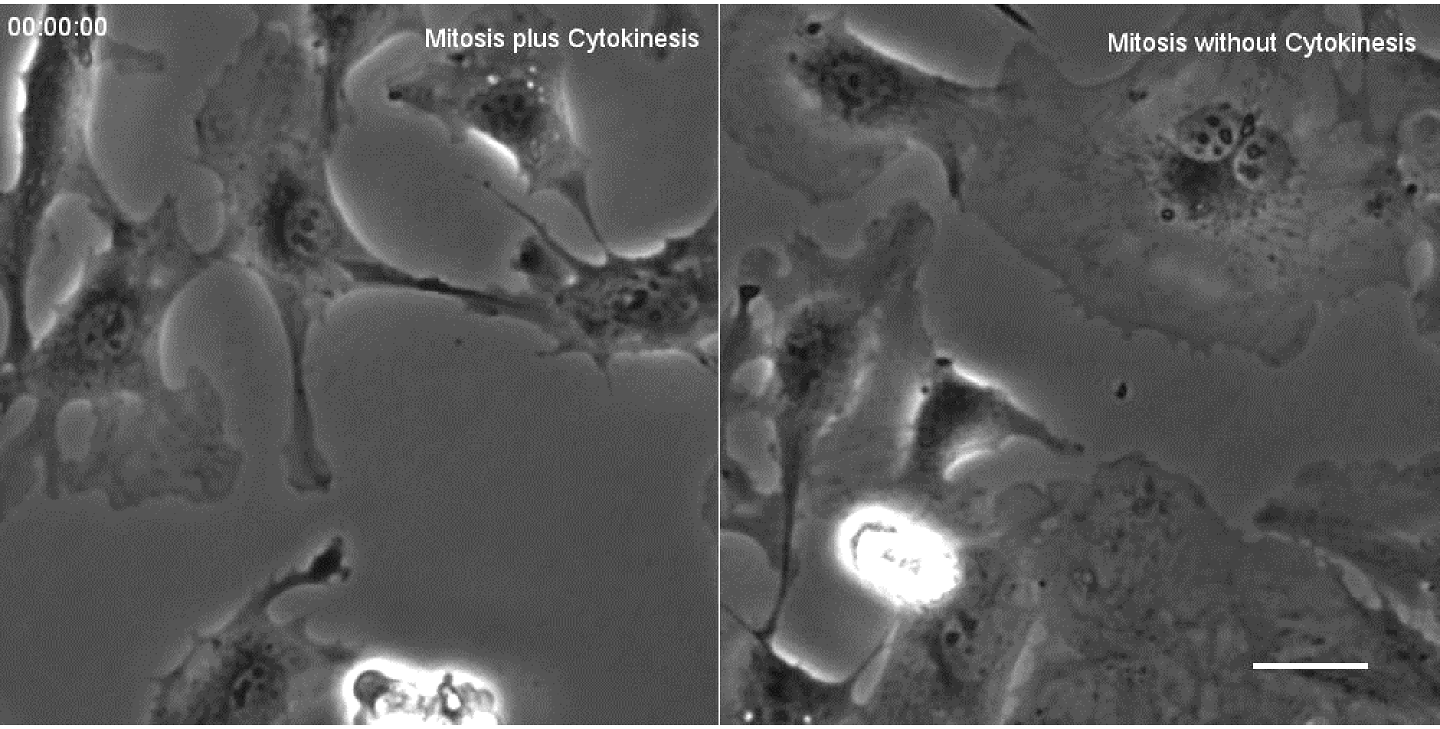

B

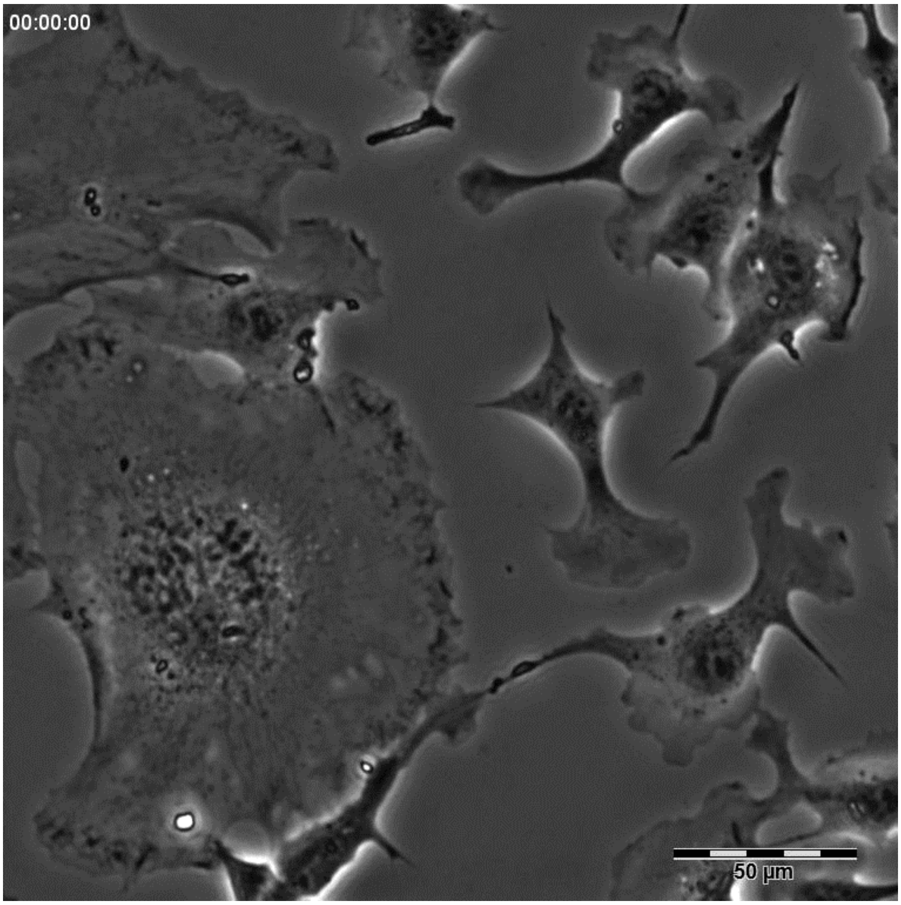

Fig. S1C

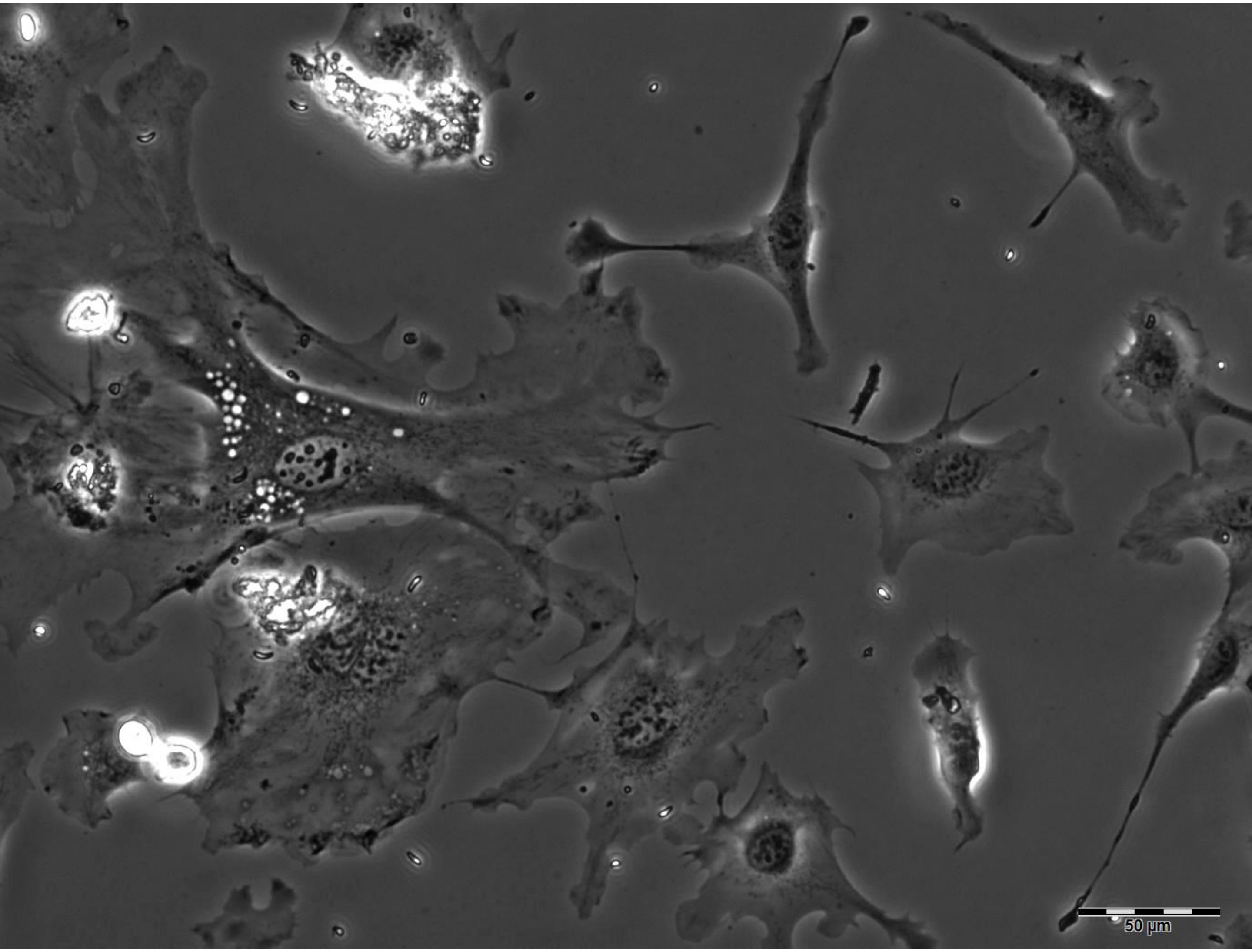

Fig. S1 : Video recording of the cytokinesis failure in *T. gondii*-infected cells.
Cytokinesis failure was recorded in parallel in non-infected and *T. gondii*-infected cells (N=3, Fig. 1E). The images were taken every 8 min during 20 h applying 40X magnification. (A) Real time videos on cytokinesis in non-infected (left panel, mitosis plus cytokinesis) and *T. gondii*-infected cells (right panel, mitosis without cytokinesis). (B) Zoom on a single infected cell that still contains two nuclei after cell division . (C)

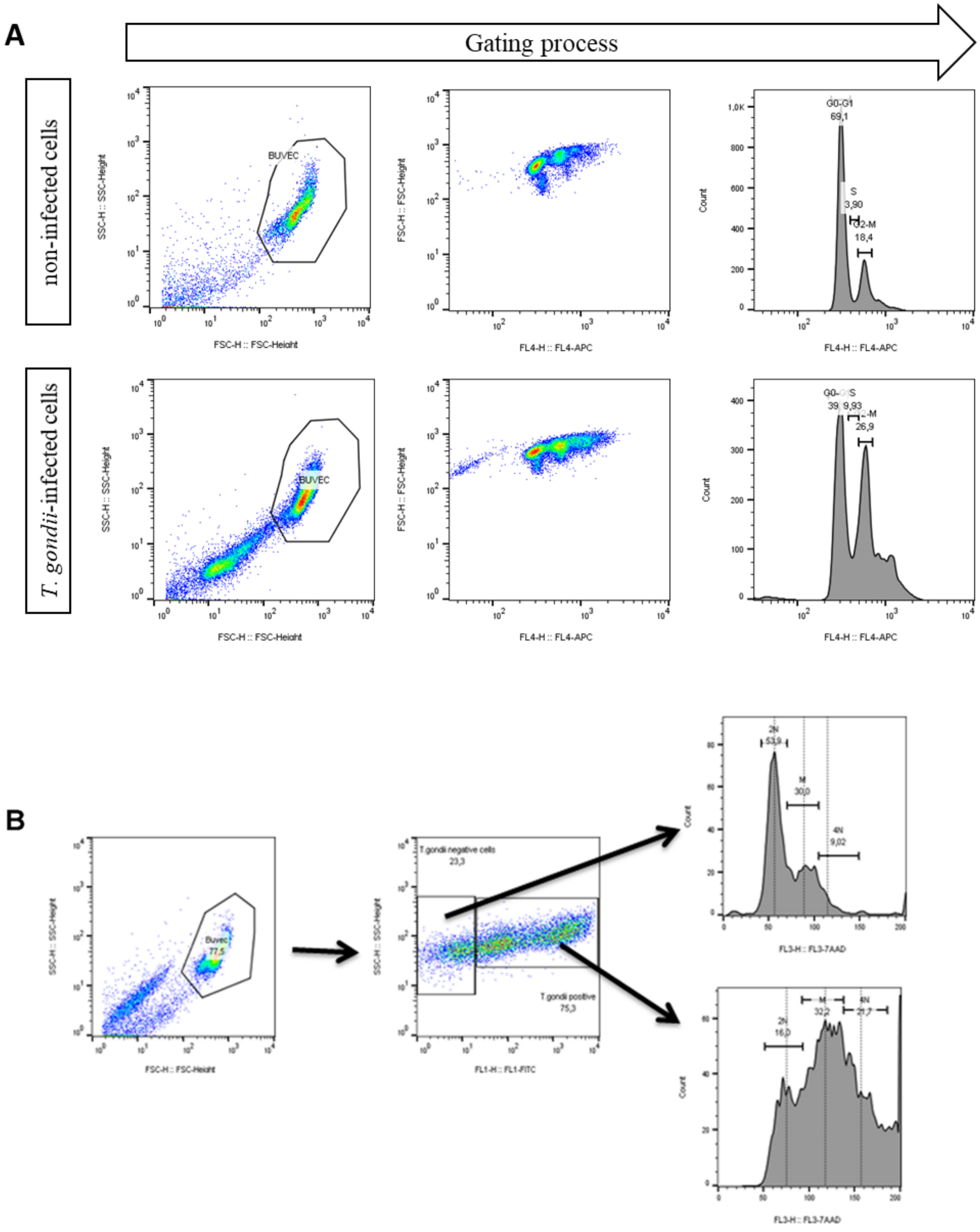

Fig. S2 : Gating strategy in FACS analysis of *T. gondii*-infected cells

(A) Gating process for the data shown in Fig. 2A. The BUVEC population was selected, and the histogram vs APC plot was used to detect the three main cell cycle phase (G0/G1-; S-, and G2/M-phase). (B) Gating process for data presented in Fig. 2B. BUVEC population was selected and afterwards, two populations were split in terms of the positive (infected cells) or negative (non-infected cells) signal for *T. gondii*-specific antibody-mediated staining. The histogram vs APC signal plot was used to detected the three main DNA populations (G0/G1-; S-, and G2/M-phase).

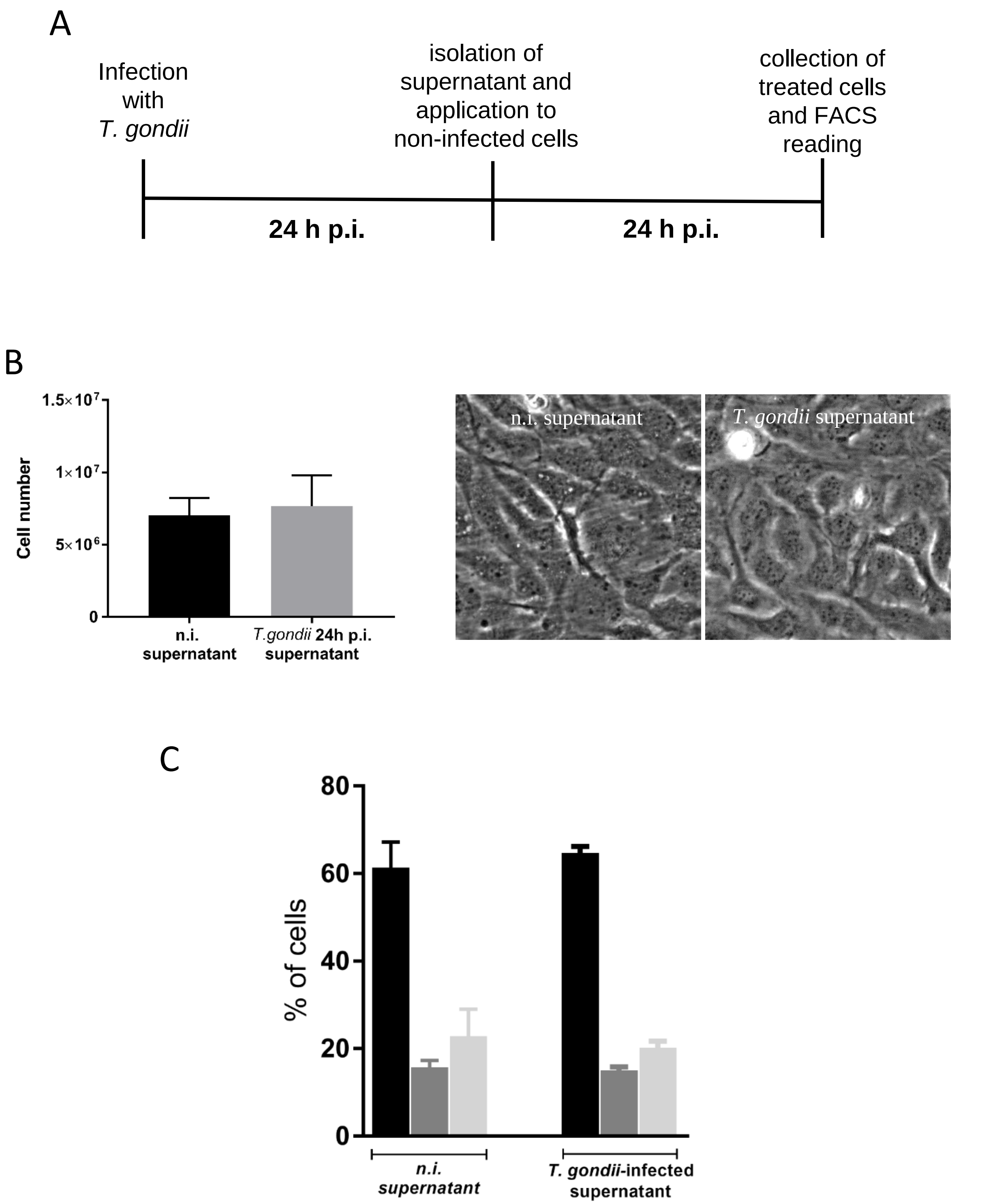

Fig. S3 : Effect of supernatants from *T. gondii*-infected host cells on the cell cycle of non-infected cells

(A) Experimental procedure: Supernatants from *T. gondii*-infected host cells (24 h p. i.) and non-infected control cells were filtered (0,2 µm filters) and applied to non-infected cells (N=4) for 24 h. Then, treated cells were detached, fixed and analysed by FACS. (B) Total number of cells in the monolayer after 24 h of treatment with supernatant. (C) FACS analysis of the G0/G1 (black bars)-, S- (dark grey bars), and G2/M-phases (light grey bars).

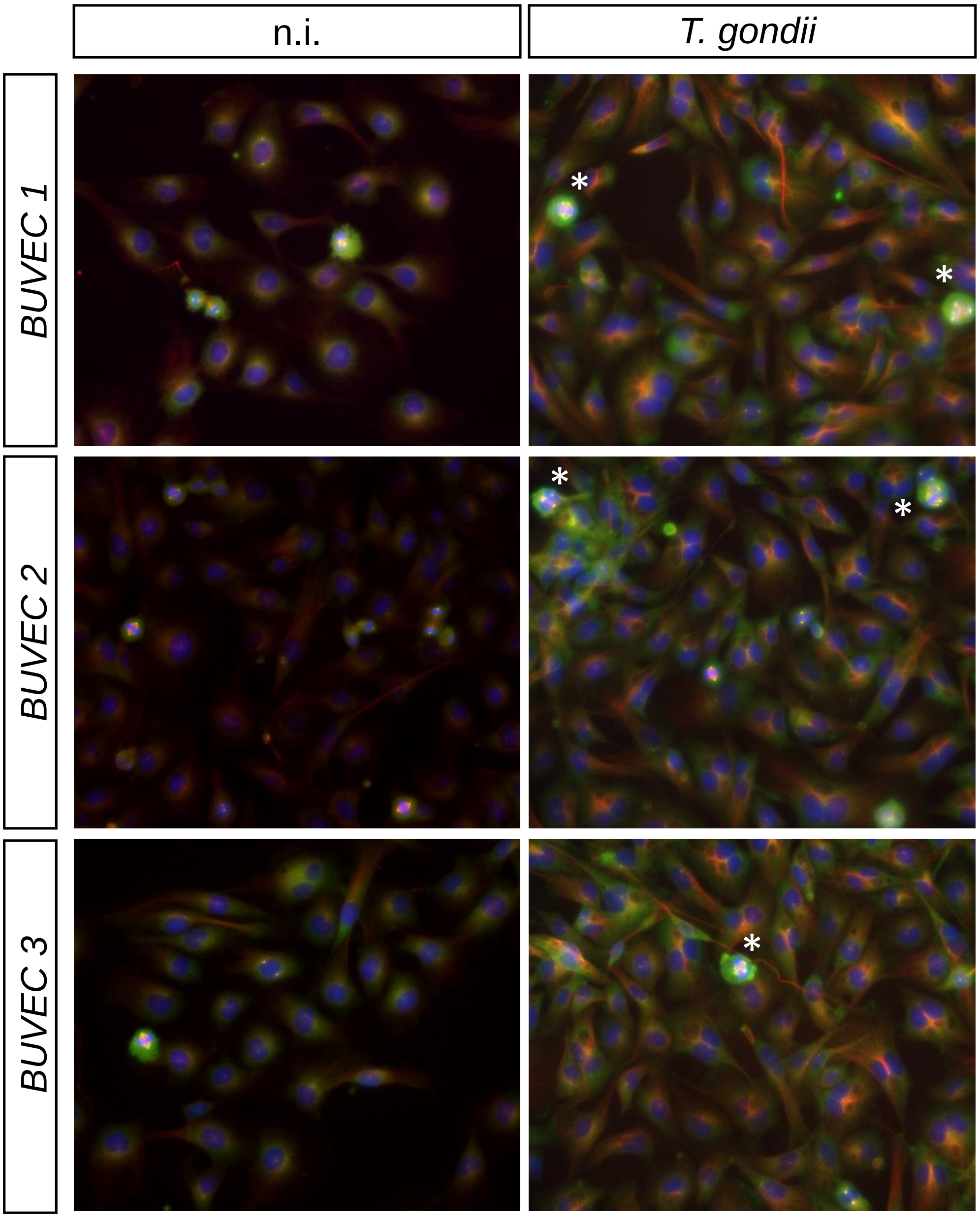

Fig. S4 : Analysis of centrosome numbers in non-infected and *T. gondii*-infected cells.

In *T. gondii*-infected BUVEC and non-infected control cells the total number of centrosomes per mitotic spindle was counted (Fig. 3). Therefore, non-infected and *T. gondii*-infected BUVEC (24 h p. i., N=3 BUVEC isolates) were stained for α- and γ-tubulin (centrosomes and mitotic spindle detection, respectively). Nuclei and chromosomes were stained with DAPI. The images were taken at 40X magnification and a total of 500 cells were counted in both conditions. Asterisks indicate cells with more than two centrosomes. The mitotic spindle structure is shown in detail in Fig. 5A and the number of centrosomes in Fig. 5B.

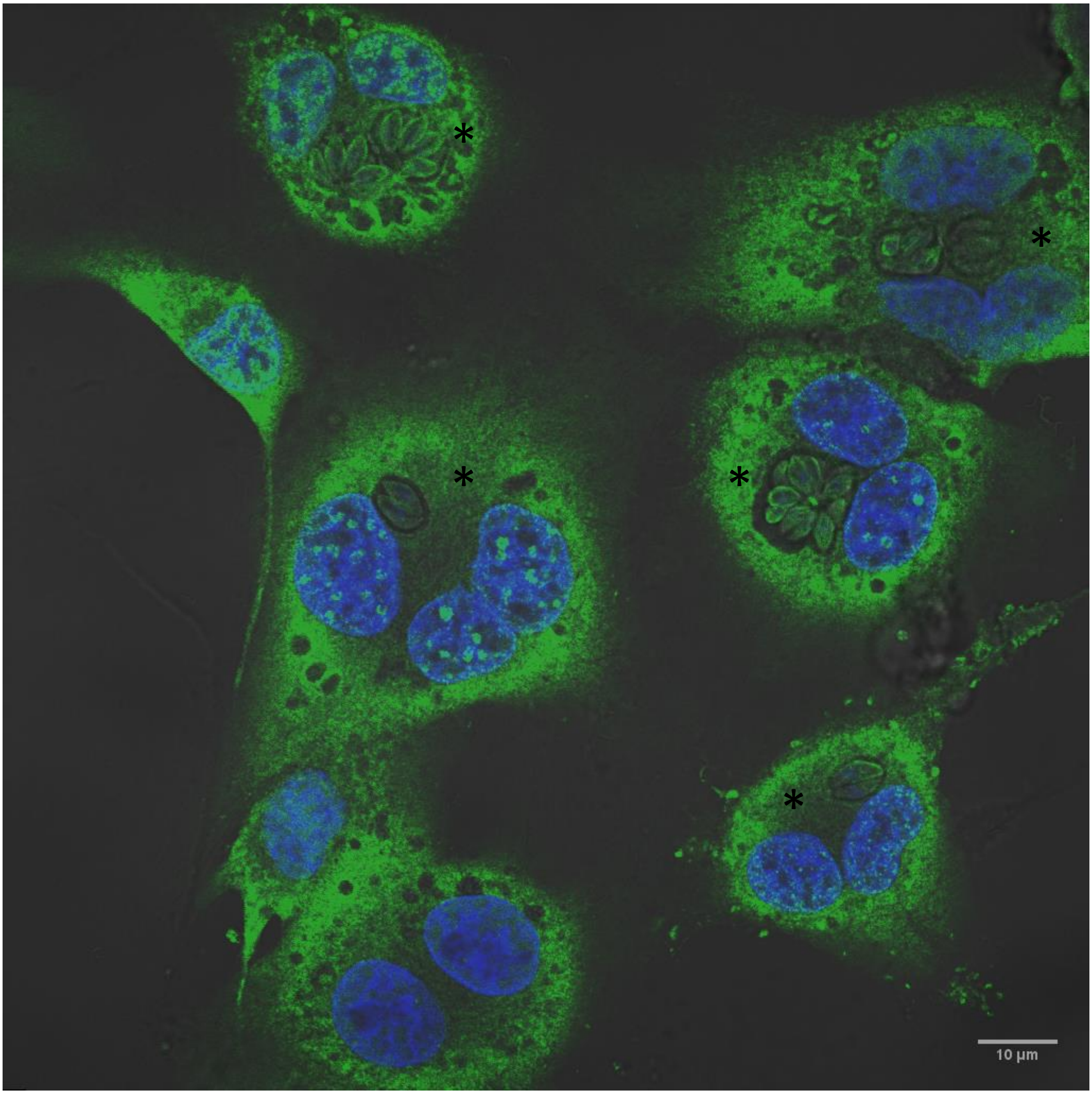

Fig. S5 : Complete vision field of *T. gondii*-infected cells stained with PCNA
Cells infected with *T. gondii* for 24 h p.i. (N=3) were stained for the PCNA. The nuclear localization of the PCNA signal was detected in detail to illustrate S-phase progression (see Fig. 6). Magnification: 63X.

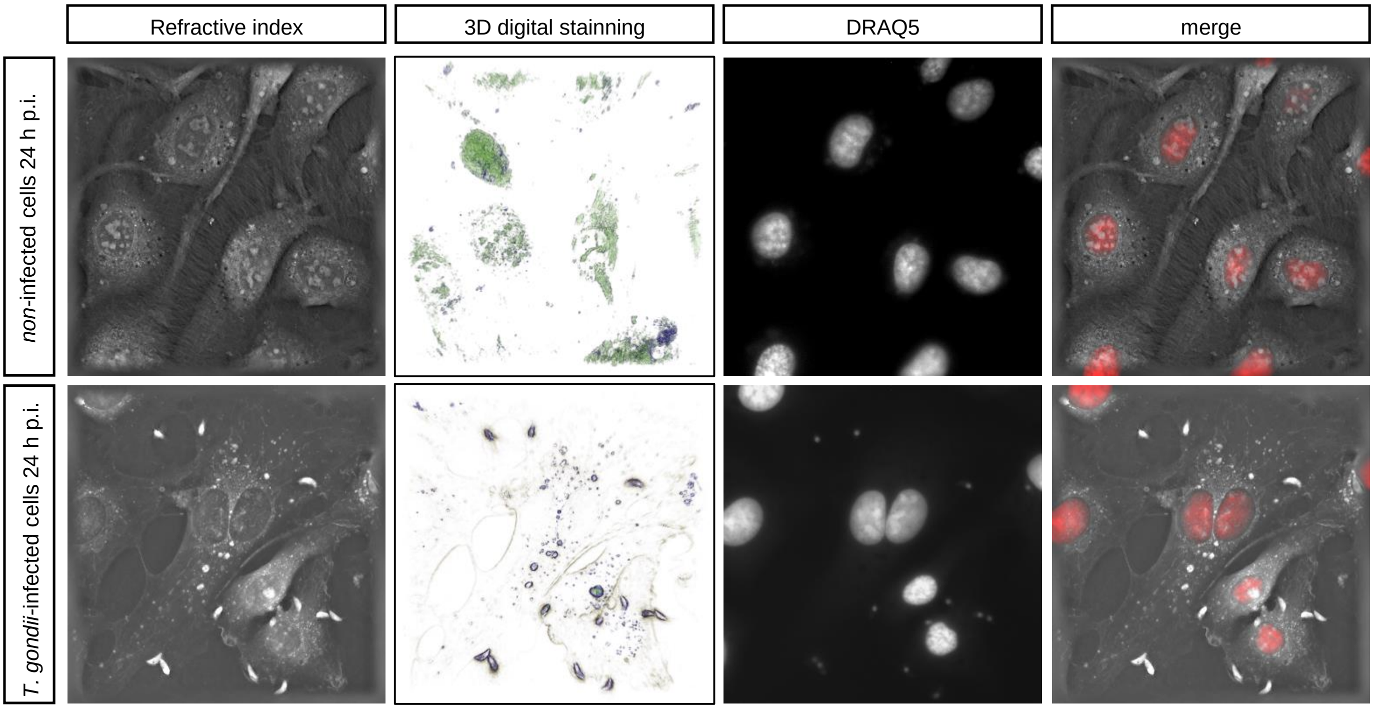

Fig. S6 : Vital staining of live binucleate *T. gondii*-infected BUVEC

Cells infected with *T. gondii* were stained with the vital stain DRAQ5 (red) for nuclei detection.
